# Preconceptual care reduces risk of spontaneous preterm birth for select high‐risk populations: A prospective study

**DOI:** 10.1002/ijgo.70340

**Published:** 2025-07-04

**Authors:** Gillian A. Corbett, Larissa Luethe, Aoife McEvoy, Maggie O'Brien, Brian McDonnell, Silvia Carlotta Rosalia, Fionnuala M. McAuliffe, Siobhan Corcoran

**Affiliations:** ^1^ National Maternity Hospital Dublin Ireland; ^2^ Preterm Birth Service National Maternity Hospital Dublin Ireland

**Keywords:** abdominal cerclage, cervical cerclage, preconception, preterm birth, preterm birth prevention, vaginal progesterone

## Abstract

**Objective:**

Prevention of preterm birth (PTB) is an urgent unmet clinical need. Preconceptual counseling for women at high‐risk of PTB offers a unique opportunity to mitigate risk of preterm birth for subsequent pregnancy. The present study aimed to examine the impact of preconceptual counseling on rates of PTB in a high‐risk population.

**Methods:**

This was a prospective study at a tertiary referral PTB Clinic of all preconceptual consultations over a 5‐year period (2018–2023, *n* = 97). Consultation data were collected. Subsequent pregnancy outcomes for women who attended for preconceptual counseling were compared to a women attending the PTB Clinic who did not attend for preconception counseling, with sensitivity analysis based on PTB risk factor.

**Results:**

A total of 97 women attended for preconception care with 47 subsequent pregnancies, of which 62/97 (63.9%) had a prior PTB or mid trimester loss (MTL). A total of 35/97 (36.0%) had cervical surgery. After consultation, 34/97 (35.0%) patients chose abdominal cerclage, 15/97 (15.5%) chose elective cervical cerclage in pregnancy and 48/97 (49.5%) opted for reassessment in early pregnancy. Pregnancy outcomes were recorded for 165 women attending the PTB Clinic; this included 47 women who had received preconceptual counseling and 118 who did not receive preconceptual counseling. Women with preconceptual care were significantly more likely to have an abdominal cerclage (17/47, 36.2% vs. 4/118, 3.4%, *P* < 0.001). There was no difference in preterm birth <37 or <34 weeks or mid‐trimester loss with preconceptual counseling for any risk factor criteria. However, for high‐risk patients (history of extreme PTB [<28 week], mid‐trimester loss [14–23 weeks] or recurrent sPTB) preconceptual counseling was associated with a significant reduction in early PTB <32 weeks (0/19, 0.0% vs. 6/32, 18.8%, *P* = 0.044), associated with significantly higher rate of abdominal cerclage (5/19, 26.3% vs. 0/0, 0.0%, *P* = 0.003).

**Conclusion:**

Preconceptual counseling is an effective intervention to reduce risk of early preterm birth for select high‐risk populations.

## 1 INTRODUCTION

Preterm birth, defined as birth before 37 weeks' gestation, is the leading global cause of mortality for children under five.^1^ Spontaneous preterm birth (sPTB) accounts for most preterm births. Preterm birth is associated with significant health, psychological and financial cost for the individual, their family and society as a whole.^2^, ^3^, ^4^ Despite this, strategies to effectively predict and prevent spontaneous preterm birth are lacking. Although advances in research surrounding progesterone therapy, cervical and abdominal cerclage,^5^ the global rate of preterm birth has not decreased in the last decade.^6^ Preterm birth remains the single greatest contributor to loss of human capital of any disease across the human lifecourse.^7^

Preconceptual care is defined by the WHO as interventions provided before pregnancy to promote the health and wellbeing of women and families, as well as improved pregnancy and child‐health outcomes.^8^ Preconceptual counseling has many acknowledged benefits for those at risk of preterm birth including positive patient experience and enhanced patient knowledge.^9^, ^10^ Qualitative work with experts by experience in preterm birth shows that pre‐conceptual counseling, anticipation of risk and an active approach to risk reduction can improve sense of control, mitigate the trauma of an unanticipated spontaneous preterm birth. This can improve the psychological burden for parents in subsequent pregnancy.^11^ Preconceptual counseling is associated with improved pregnancy outcomes including greater disease control and reduced rates of adverse pregnancy outcomes including preterm birth in patients with chronic medical disorders.^12^, ^13^ Yet, impact of preconceptual counseling on rates of PTB in those with risk factors is not yet well established.^14^

Preconceptual prevention of preterm birth may encompass several strategies. Preconceptual supplementations with folate and omega 3 reduces risk of preterm birth.^5^, ^15^ Mitigation of predisposing factors such as smoking cessation is associated with reduced risk of PTB.^16^ Most conventional preterm prevention therapies such as vaginal progesterone, cervical cerclage and cervical pessary are not offered until the end of the first trimester of pregnancy. Abdominal cerclage can be offered either in pregnancy or ahead of pregnancy with judicious population selection. Recent cohort studies have found that laparoscopic pre‐pregnancy abdominal cerclage may be associated with higher rates of full‐term birth.^17^ The Born Too Soon movement 2013 report recommends optimization of medical conditions, nutritional factors, mental health, smoking patterns, sexually transmitted infections and intimate partner violence for all women of reproductive age to reduce population risk of PTB.^18^ It recommends evaluation of impact of preconception care programs on rates of preterm birth but since this report, there have been limited data specifically dedicated to preconceptual preterm birth prevention care.

At the Preterm Birth Clinic of this center, formal preconceptual care has been delivered for women at risk of PTB since 2012, with service explansion from 2018. The present study examined the structure, assessment and management of these consultations, as well as impact on preterm birth rates in subsequent pregnancies.

## 2 MATERIALS AND METHODS

This was a prospective quasi‐experimental study of all preconceptual counseling consultations and subsequent pregnancy outcomes at the Preterm Birth Clinic at a tertiary referral maternity center, Dublin, Ireland over a 5‐year period (2018–2023). The center is a tertiary‐level university maternity hospital. Inclusion criteria was any woman who attended the Preterm Birth Clinic for pre‐pregnancy counseling with risk factors including prior PTB, spontaneous mid‐trimester loss (sMTL), recurrent PTB or significant cervical surgery such that the cervix is noted to be short on clinical examination. These patients are referred for counseling from a variety of sources where their risk of PTB was deemed to be significant: internal and external obstetric referral, bereavement services, neonatology colleagues, colposcopy colleagues, or referral from general practice or patient themselves. Preterm birth categories are defined according to the WHO as extremely preterm (less than 28 weeks), very preterm (28 to less than 32 weeks), late preterm (32–37 weeks),^7^ with mid‐trimester loss defined as delivery between 14^+0^ and 22^+6^ weeks gestation (inclusive). The threshold of viability in this unit is 23^+0^ weeks gestation, differentiating late second trimester loss and extreme early preterm birth.

**FIGURE 1:**
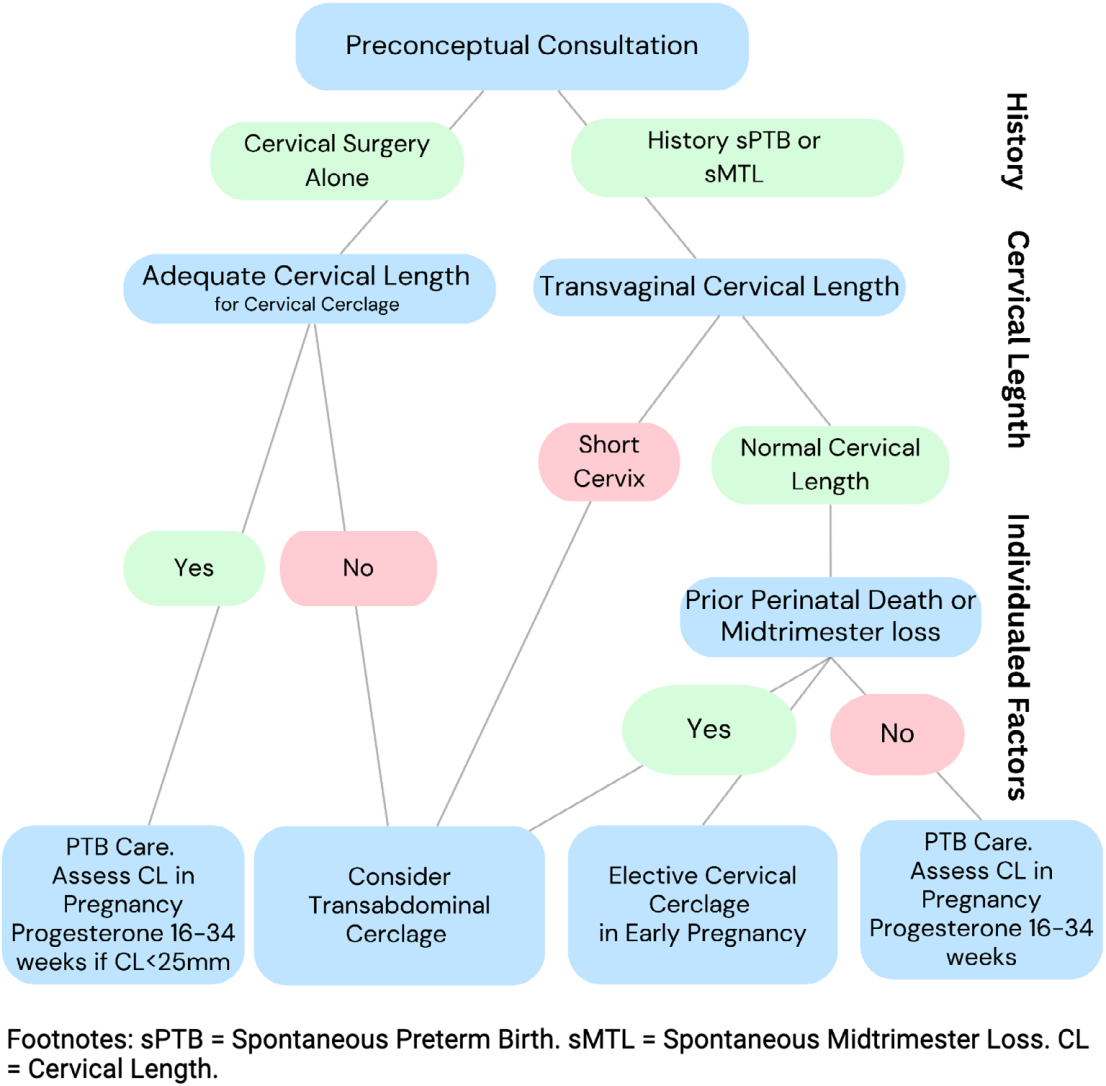
Preconceptual consultation decision tree, illustrating key factors in decision making including exact preterm birth history, pre‐pregnancy cervical length and individualized situational factors.

The decision‐making tree used for preconceptual consultations is presented in Figure 1. Preconceptual counseling was conducted by a maternal‐fetal medicine consultant with special interest in preterm birth. Content of preconceptual counseling is described in detail in Appendix S1. Counseling was conducted over a one 30‐min consultation, with the option for a second or third visit if additional follow up discussion is required. Factors important for decision making included significant history of sPTB/sMTL and cervical length on transvaginal ultrasound. Women with a history of prior sMTL, perinatal death resulting from sPTB before 28 weeks or recurrent sPTB are deemed particularly high‐risk, with the first two criteria also experiencing the trauma associated with perinatal loss. This is a major factor in subsequent pre‐pregnancy decision making. At pre‐pregnancy consultation, four management options are outlined to the patient, and a joint decision is made based on individualized risk assessment of sPTB. Management options include:

1. Preterm Birth Clinic care with cervical length surveillance, reserving progesterone or cerclage for a cervical length <25mm.
2. Preterm Birth Clinic care with progesterone (if history of sPTB or cervical length <25 mm in pregnancy) and consideration for cerclage if cervical length <25 mm on serial cervical scanning.
3. Preterm Birth Clinic care with elective cervical cerclage in early pregnancy, based on history and pre‐pregnancy cervical length.
4. Abdominal cerclage pre‐pregnancy.

For those with a subsequent pregnancy, pregnancy outcomes were analyzed and compared to a contemporary sPTB cohort of consecutive patients attending the Preterm Birth Clinic (2021–2023) who did not receive preconceptual counseling. The criteria for identification of these women were the same as for preconceptual counseling, being either a prior preterm birth or significant cervical surgery and difference in risk factor profile including high‐risk criteria (prior MTL, PTB <28 weeks or recurrent PTB) was examined between groups. Maternal demographics, preterm birth risk factor profile, sPTB prevention strategy and pregnancy outcomes were compared between pregnancy cohorts. Cervical length is measured at booking for all women, except those with a pre‐pregnancy abdominal cerclage in place.

At this Preterm Birth Clinic, booking occurs at 10–12 weeks' gestation and cervical length assessment and individualized preterm birth risk assessment is performed, after screening and treatment of vaginal infection. Vaginal progesterone (200 mg nocte until 34 weeks' gestation) is recommended for anyone with prior sPTB or sMTL or if cervical length is <25 mm. Cervical cerclage is considered if cervical length is <25 mm at booking or at subsequent serial cervical length. A cervical pessary is considered for those with a very short cervix where there is technical challenge associated with placement of cervical cerclage or disputed benefit of cervical cerclage. Abdominal cerclage is considered in the first trimester if there is history of recurrent sPTB/MTL refractory to cervical cerclage. All women having cervical cerclage, abdominal cerclage or pessary for short cervix are commenced on vaginal progesterone, if not already commenced. The cervical cerclage or pessary is removed at 36 weeks' gestation or sooner if labor or preterm rupture of membranes. Serial cervical length assessment is performed every 3 weeks from booking until 28 weeks. If a cerclage or abdominal cerclage, or cervical pessary is in place in, serial cervical length assessment is not performed. Vaginal progesterone is coadministered if there is a significant history of sPTB (recurrent MTL or sPTB, particularly if perinatal loss). Women with abdominal cerclage are delivered by prelabour cesarean section at 38 weeks' gestation.

Descriptive statistics were used to analyze all preconceptual consultations. To compare pregnancy cohorts, data were assessed for normality and log transformed where non‐normal data was present. Normally distributed continuous outcomes were compared using an independent *t*‐test and expressed using mean and standard deviation (SD). Non‐normally distributed continuous outcomes were compared using a Wilcoxon rank test and expressed using median and interquartile range (IQR). Categorical variables were compared using the chi‐square test. Sensitivity analysis was performed based on risk factor profile for cervical surgery alone and for very high risk of preterm birth, defined as prior spontaneous mid‐trimester loss, preterm birth before 28 weeks or recurrent sPTB. Statistical significance was taken as a *P* value less than 0.05. Given the exploratory nature of the study and relatively small sample size, correction for multiple testing was not performed.^19^, ^20^ All statistical analysis was performed using R studio (R version 4.3.2 [2023‐10‐31]). Ethical approval was granted by the Institutional Review Board (study ID E15.2022).

## 3 RESULTS

In the 5‐year period, 97 women attended for preconceptual counseling (Table 1). Source of referral for preconceptual counseling included internal colposcopy (25/97, 27.5%), internal neonatology (6/97, 6.6%), internal obstetric (38/97, 39.2%), external obstetric (9/97, 9.9%), general practitioners (8/97, 8.8%), fertility centers (8/97, 8.8%) and self‐referral from patients (3/97, 3.3%). There was a prior history of sPTB or sMTL in 62/97 (64%), with 36/40 (36.0%) having a history of cervical surgery alone. Of the women with previous sPTB/MTL, 20 had significant risk factors prior to the sPTB/MTL including multiple pregnancy, fully dilated cesarean section or previous cervical surgery. Median (IQR) cervical length at pre‐pregnancy consultation was short at 19.0 (13.0) mm. After consultation and joint decision making (Figure 1), 48/97 (49.5%) chose reassessment in early pregnancy and consideration of vaginal progesterone and cerclage, 15/97 (15.5%) chose elective cerclage in early pregnancy and 34/97 (35.0%) chose pre‐pregnancy abdominal cerclage.

**TABLE 1:** Maternal characteristics at pre‐pregnancy consultation between 2018 and 2023 at the Preterm Birth Clinic (*n* = 97).

	Pre‐pregnancy consultations (*n* = 97)
Maternal age^a^	33.5 (6.0)
BMI^a^	26.4 (10.1)
Parity (multiparous)	46 (56.8%)
Ethnicity (non‐Caucasian)	13 (13.4%)
Educational attainment (3rd level complete)	62 (63.9%)
Risk factor for sPTB	
sPTB/MTL alone	42 (43.4%)
sPTB/MTL with major RF (Cx surgery, FDCS, multiple pregnancy)	20 (26.6%)
Cervical surgery alone	35 (36.0%)
Prior preterm birth	
Prior sMTL (14^+0^–22^+6^ weeks)	21 (21.7%)
Prior sPTB (23^+0^–27^+6^ weeks)	23 (23.7%)
Prior sPTB (28^+0^–31^+6^ weeks)	33 (34.0%)
Prior sPTB (32^+0^–36^+6^ weeks)	9 (9.3%)
Prior perinatal death or periviable loss	24 (24.7%)
Cervical length at pre‐pregnancy consultation	19.0 (13.0)
Selected pathway to prevention	
Pre‐pregnancy abdominal cerclage	34 (35.0%)
Elective cervical cerclage early pregnancy	15 (15.5%)
PV progesterone and reassessment in early pregnancy ± cerclage	48 (49.5%)

*Note*: BMI, calculated as weight in kilograms divided by the square of height in meters.

Abbreviations: BMI, body mass index; Cx, cervical; FDCS, fully dilated cesarean section; IQR, interquartile range; MTL, mid‐trimester loss; PV, per vaginum; RF, risk factors; sPTB, spontaneous preterm birth.

^a^ Non‐normal variable, expressed as median (IQR).

**FIGURE 2:**
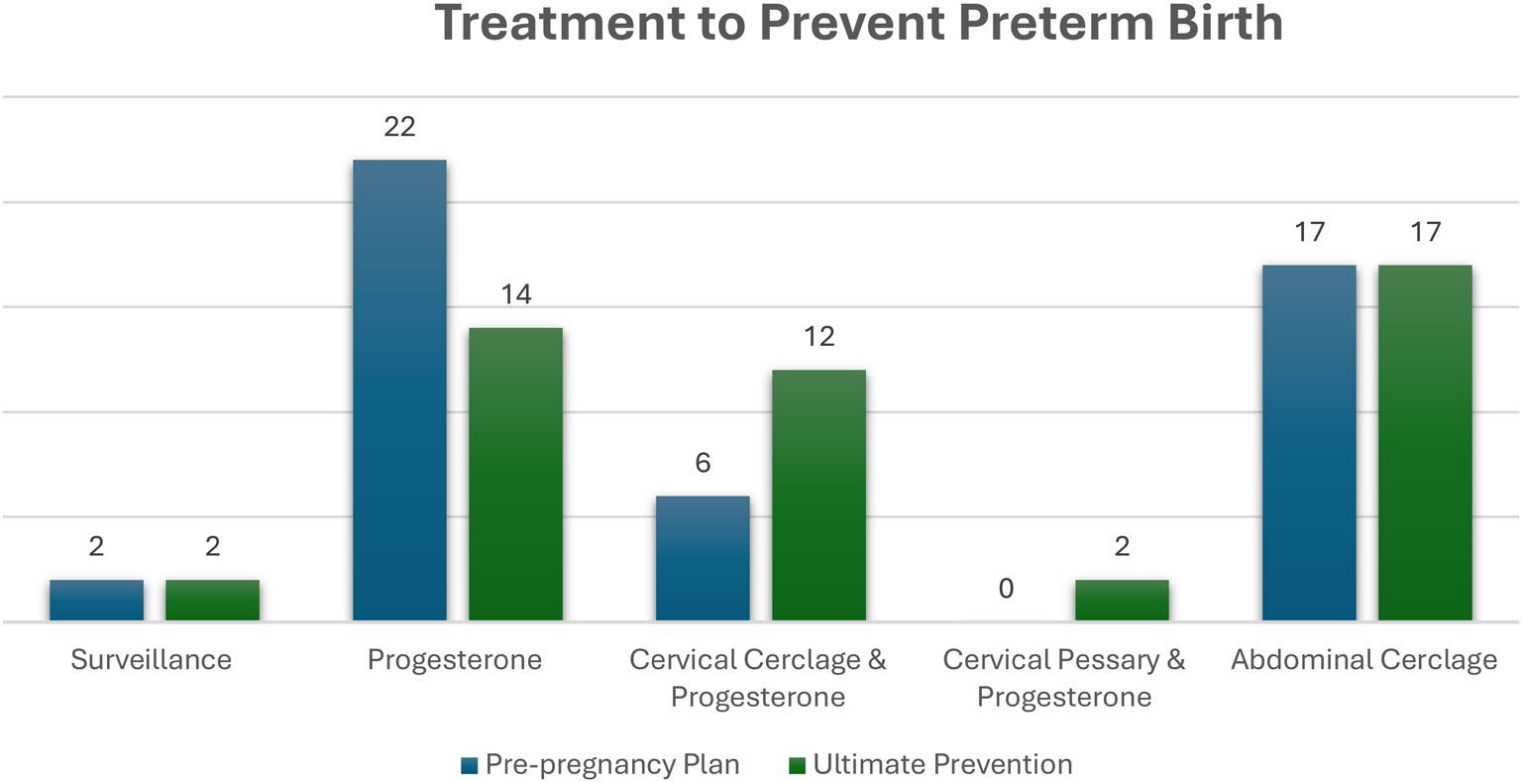
Interventions selected at pre‐pregnancy consultation versus ultimate preterm birth prevention required in pregnancy.

Of the women who attending preconceptual counseling, 47/97 (48.5%) had a subsequent pregnancy. Management at pre‐pregnancy counseling versus ultimate sPTB prevention management in pregnancy is presented in Figure 2. These pregnancies were compared to comparison pregnancy cohort with similar risk profile who did not have preconceptual counseling, with no significant difference in risk factors for preterm birth between the two cohorts. Pregnancy outcomes for 165 women were analyzed, 47 who attended for preconceptual counseling and 118 who did not. Women in pregnancy after preconceptual counseling were slightly younger and less multiparous than the comparison group (Table 2) with no difference in BMI, ethnicity or educational attainment. There was no difference in sPTB risk factor profile between the pregnancy cohorts, including similar prevalence of high‐risk populations between both groups (with a history of sMTL, sPTB before 28 weeks or recurrent sPTB). Cervical length at booking was similar between groups, where cervical length was not measured for anyone with a pre‐pregnancy abdominal cerclage.

**TABLE 2:** Maternal characteristics and subsequent pregnancy outcome pre‐pregnancy consultation versus those without pre‐pregnancy consultation in women with high risk of spontaneous preterm birth.

	Pregnancy after pre‐pregnancy consultation (*n* = 47)	sPTB‐risk group without preconceptual counseling (*n* = 118)	*P* value
Maternal age^a^	33.0 (3.5)	35.0 (5.0)	0.002
BMI^a^	24.9 (9.3)	26.4 (6.6)	0.136
Parity (multiparous)	27 (57.4%)	93 (78.8%)	0.012
Ethnicity (Non‐Caucasian)	5 (10.6%)	17 (14.4%)	0.697
Educational attainment (3rd level complete)	35 (74.5%)	83 (70.3%)	1.000
Risk factor for sPTB			
sPTB/MTL alone	23 (48.9%)	61 (51.7%)	0.439
sPTB/MTL with predisposing RF	7 (14.9%)	14 (11.9%)	0.603
Cervical surgery alone	17 (36.2%)	43 (36.4%)	0.981
High‐risk populations	19 (40.4%)	32 (27.1%)	0.096
Cervical length at booking (cm)	2.9 (1.1)	3.1 (0.7)	0.601
Ultimate prevention treatment for sPTB			
Surveillance alone	2 (4.3%)	34 (28.8%)	0.001
Progesterone	14 (29.8%)	49 (41.5%)	0.164
Cervical cerclage and progesterone	12 (25.5%)	21 (17.8%)	0.266
Cervical pessary and progesterone	2 (4.3%)	10 (8.5%)	0.351
Abdominal cerclage	17 (36.2%)	4 (3.4%)	<0.001
Gestation at delivery^a^	38.1 (1.8)	38.9 (2.6)	0.041
Term birth ≥37 weeks	40 (85.1%)	99 (83.9%)	0.848
Any sPTB/MTL (14–37 weeks)	7 (14.9%)	19 (16.1%)	0.165
sMTL 14–23 weeks	3 (6.4%)	3 (2.5%)	0.234
Extreme PTB 23–28 weeks	0 (0.0%)	4 (3.4%)	0.201
Early PTB 23–32 weeks	0 (0.0%)	7 (5.9%)	0.088
Late PTB 32–37 weeks	4 (8.5%)	9 (7.6%)	0.849

*Note*: BMI, calculated as weight in kilograms divided by the square of height in meters. High‐risk population = previous spontaneous mid‐trimester loss or preterm birth before 28 weeks, or recurrent sPTB. Categorical variables compared with Chi‐square test. Expressed as *n* (% of denominator). *P* value <0.05 was statistically significant. Na, comparison not performed due to zero values.

Abbreviations: BMI, body mass index; IQR, interquartile range; MTL, mid‐trimester loss; NA, not applicable; PTB, preterm birth; RF, risk factors; sPTB, spontaneous preterm birth.

^a^ Non‐normal distribution—compared using Wilcoxon rank test. Expressed as median (IQR).

Women without preconceptual counseling were more likely to have surveillance without need for progesterone or cervical cerclage (34/118, 28.8% vs. 2/47, 4.3%, *P* = 0.001), and were less likely to have abdominal cerclage (4/118, 3.4% vs. 17/47, 36.2%, *P* < 0.001). Gestation at delivery was significantly lower in the preconceptual care group (median, IQR 38.1.[1.8] vs. 38.9 [2.6], *P* = 0.041). There was no significant difference in rates of term birth, or preterm birth or mid‐trimester loss of any category. No sPTB before 32 weeks' gestation occurred for women who had preconceptual care prior to pregnancy (Table 2).

Sensitivity analysis was performed for all women with very high‐risk history (*n* = 51, Table 3). These women were significantly more likely to have abdominal cerclage in pregnancy (5/19, 26.3% vs. 0/32, 0.0%, *P* = 0.003). There was no significant difference in gestation at delivery or rate of full‐term birth. However, there was a significant reduction in the rate of sPTB before 32 weeks in women who had preconceptual counseling (0/19, 0.0% vs. 6/32, 18.8%, *P* = 0.044). On regression analysis of impact of intervention on gestation at delivery and adjusted for maternal confounders and preterm birth treatment, preconceptual counseling did not significantly alter gestation at delivery (ß coefficient 0.49, 95% CI: −2.11, 3.03). Of the high‐risk women with sMTL after preconceptual care (*n* = 3), all had cervical cerclage in pregnancy with mid‐trimester loss refractory to cervical cerclage (delivery gestation ranging from 14 to 21 weeks of pregnancy). All three of these women went on to have abdominal cerclage after this loss and had a subsequent full‐term delivery. For women with previous excisional cervical surgery but no history of preterm birth (*n* = 60), preconceptual counseling did not impact sPTB or sMTL rates at any gestation.

**TABLE 3:** Sensitivity analysis for select high risk population (with prior spontaneous mid‐trimester loss or preterm birth before 28 weeks, or recurrent sPTB).

	Preconceptual care (*n* = 19)	sPTB‐risk group without preconceptual counseling (*n* = 32)	*P* value
Ultimate prevention treatment for sPTB			
Progesterone	7 (36.8%)	15 (46.9%)	0.486
Cervical cerclage and progesterone	7 (36.8%)	17 (53.1%)	0.264
Abdominal cerclage	5 (26.3%)	0 (0.0%)	0.003
Gestation at delivery^a^	38.0 (11.0)	38.0 (2.7)	0.984
Term birth ≥37 weeks	14 (73.7%)	20 (62.5%)	0.413
Any sPTB/MTL (14–37 weeks)	5 (26.3%)	12 (37.5%)	0.413
sMTL 14–23 weeks	3 (15.8%)	3 (9.4%)	0.492
Extreme PTB 23–28 weeks	0 (0.0%)	4 (12.5%)	0.108
Early PTB 23–32 weeks	0 (0.0%)	6 (18.8%)	0.044
Late PTB 32–37 weeks	2 (10.5%)	3 (9.4%)	0.894

*Note*: Categorical variables compared with Chi‐square test. Expressed as *n* (% of denominator).

Abbreviations: IQR, interquartile range; MTL, mid‐trimester loss; PTB, preterm birth; sMTL, spontaneous mid‐trimester loss; sPTB, spontaneous preterm birth.

^a^ Non‐normal distribution—compared using Wilcoxon rank test. Expressed as median IQR.

## 4 DISCUSSION

The present study details preconceptual consultations for women at risk of sPTB and found that for a select high‐risk subgroup, preconceptual counseling significantly reduced the risk of sPTB before 32 weeks. Women receiving preconceptual counseling for risk of sPTB were more ethnically diverse and overweight than the general hospital population^21^ and had a short cervix at consultation. Almost two‐thirds had a prior sPTB or MTL, with 45% having a birth before 28 weeks and 24.7% having prior perinatal death or periviable loss. There was a high rate of uptake of pre‐pregnancy abdominal cerclage. Most women did not change ultimate prevention of preterm birth management in pregnancy. Of women with preconceptual counseling, those who had a subsequent pregnancy were slightly younger, normal weight, less diverse and had a higher educational attainment.

Many studies have found reduced risk of preterm birth with preconceptual care and optimizing nutritional factors, tobacco exposure, summarized by a recent FIGO checklist for preconceptual care.^22^ These studies are based mainly on data from general obstetric populations. Specific to preterm birth cohorts, a review of 92 pre‐pregnancy consultations in a New Zealand preterm birth service found similar rate of opting for pre‐pregnancy cerclage (31/92, 33.7%,) but were a combination of abdominal cerclage high transvaginal cerclage with circumferential dissection.^23^ There was a similar rate of subsequent pregnancy (39/92, 42.4%) but pregnancy outcomes were not detailed.^23^ Review of the literature found a lack of contemporary data examining outcomes after preconception counseling for women at high‐risk of preterm birth, underlining the importance of data presented in this study.

As highlighted in many documents on preterm birth prevention, population selection is paramount.^24^ This study found that preconceptual care did not reduce preterm birth rates for all patients. For women with history of cervical surgery but no history of preterm birth, preconceptual care does not reduce preterm birth rate. However, for women with a particularly high‐risk histories, preconceptual counseling is associated with a significant reduction in rate of sPTB under 32 weeks compared to a control cohort with similar risk factor profile who did not receive pre‐pregnancy counseling. This reduction appears to be actioned through the uptake of pre‐pregnancy abdominal cerclage, which is significantly higher in the pre‐pregnancy consultation group. Rates of recurrence of preterm birth are highest with preterm birth before 28 weeks or mid‐trimester loss, ranging from 21% to 27%.^25^, ^26^, ^27^ Indeed, data presented here found that for women with sPTB <28 weeks, mid‐trimester loss or recurrent sPTB, the recurrence rate was 37.5% in the subsequent pregnancy (Table 3). As such, effective interventions such as preconceptual counseling to reduce subsequent preterm birth hold significant potential for impact both on health of the mother and child, as well as the healthcare facility and wider society.

This study identifies a high‐risk subgroup of women (prior sPTB <28, sMTL or recurrent sPTB) in whom preconceptual care reduces risk of early PTB <32 weeks' gestation. These women will benefit from referral to a preterm birth clinic or local obstetric with equivalence expertise ahead of subsequent pregnancy. In our center, this consultation is typically timed for 6–12 months after the preterm birth, which allows for recovery from birth and pregnancy, neonatal care and events to evolve and spans the minimum recommended interpregnancy interval of 6 months. This study assists in standardizing referral criteria for pre‐pregnancy care for those at significant risk of preterm birth. This has a role in promoting access equity by offering pre‐pregnancy consultation to all, including those who often miss the opportunity for pre‐pregnancy optimization including women of non‐European descent, higher deprivation scores and lower socioeconomic status.

Limitations of this study include the small cohort, with approximately twenty pre‐pregnancy consultations are performed per year at our Preterm Birth Clinic. This sample size limits data modeling and extrapolation, particularly for subgroups and adjusting for confounders The possibility of chance findings because of multiple testing should be considered and our results should be interpreted in the context of this. Future multicenter studies with larger study samples and prospective concurrent control groups are required to improve power and validation of findings of these single center findings. Another limitation is absence of confounders including socioeconomic group, stress factors, smoking and chronic comorbid diseases, which are known modifiable factors for preterm birth. They are included in preconceptual counseling discussion and would be salient to include in any future analysis. These at‐risk preterm birth cohorts have high uptake of cervical and abdominal cerclage, which impacts subsequent pregnancy outcomes and lengths subsequent pregnancy. Any impact of preconceptual care on pregnancy outcome is likely to be acting through these interventions but small numbers mean regression analysis controlling for these factors is challenging. There is real value in national registries such as the UK Preterm Birth Network to pool data and outcomes for preconceptual care in preterm birth cohorts.^28^

## 5 CONCLUSIONS

Preconceptual counseling is an effective intervention to reduce risk of early spontaneous preterm birth for select high‐risk populations. Patients with prior spontaneous preterm birth before 28 weeks, spontaneous mid‐trimester loss or recurrent spontaneous preterm birth should be referred for preconceptual counseling ahead of a subsequent pregnancy.

## AUTHOR CONTRIBUTIONS

The first and senior authors devised the concept and study design. Analysis was by first and second authors. All authors contributed to data collection, curation and manuscript editing and review.

## FUNDING INFORMATION

This research was supported by funding from Science Foundation Ireland (grant 22/FFP‐A/10302) and the National Maternity Hospital Foundation charity.

## CONFLICT OF INTEREST STATEMENT

The authors have no conflicts of interest to disclose.

## Supporting information


Appendix S1.

## Data Availability

The data that support the findings of this study are available from the corresponding author upon reasonable request.
