# Supplementary material for: Preconceptual care reduces risk of spontaneous preterm birth for select high‐risk populations: A prospective study
Source: Int J Gynaecol Obstet. 2025 Jul 4;172(1):492–9. doi: 10.1002/ijgo.70340 (PMC12724032; doi:10.1002/ijgo.70340)
Supplement: Supplementary file 1 — Appendix S1. [file IJGO-172-492-s001.docx]

**Preconceptual Care Reduces Risk of Spontaneous Preterm Birth for Select High-Risk Populations: a prospective study**

**Appendix 1**

Preconceptual consultation for Preterm Birth Prevention is a highly useful clinical opportunity. The following information on timing, location and content of consultation can be used as a guide to facilitate preconceptual counselling. Consultation is performed by consultant obstetrician gynaecologists with specialist interest in preterm birth and adequate experience and expertise to counsel around risk and management of preterm birth in subsequent pregnancy.

**Timing of consultation**

- Consultation time of 20-40 minutes is usually required. If all available investigation results are sourced before the consultation, one appointment will generally be adequate for counselling. Follow up appointments can also be facilitated if patient requires time to consider management options.
- Preconceptual counselling should be offered from 6 months after a previous preterm birth. This is to facilitate physical recovery of the mother, neonatal progress of the prior preterm infant, as well as optimize interpregnancy interval of at least 6 months.
- Formal gynaecology ultrasound should be performed prior to consultation with report available for review at consultation. This scan should detail uterine structure (exclude anomaly), position (anteverted-retroverted) and pre-pregnancy cervical length.

**Location of consultation**

- Appropriate consultation space – with adequate equipment for examination including pelvic examination and transvaginal scanning
- Preconceptual consultations can be integrated with the pregnancy preterm birth service or as a separate clinical session. Care should be taken to minimise shared waiting space for women who have experienced perinatal loss and pregnant patients.

**Content of consultation**

A focused history and examination should be performed for all women with moderate-significant risk factors for spontaneous preterm birth. Suggested structure for this is included below:

| **History** | |
| --- | --- |
| **Patient Details** | Age. Gravity. Parity. |
| **Preterm Birth Risk Factor** | Describe specific preterm birth risk factor(s).  Include specific details including year, preceding events, complications, and all results of investigations:   - Placental histology - Microbiology - Post-mortem results - Mid-trimester loss work-up – thrombophilia screen, karyotyping |
| **Cervical Surgery** | Describe all excisional procedures (LLETZ, Cone, Coagulation, Other) including:   - year, location - Depth of each excision if known from histopathology report   Most recent smear and next due |
| **Obstetric history** | Full obstetric history of all pregnancies, cognisant of any caesarean sections in labour or cervical dilatation in early or mid-pregnancy |
| **Gynae History** | Include any relevant gynaecological history such as hysteroscopic surgery, uterine anomaly or other.  Mention ideal family size and plans for postnatal contraception (if appropriate) |
| **Medical history** | Full medical history |
| **Medications** | Include current medications and medications prior to pregnancy |
| **Drug Allergies** | Particularly antibiotic allergies, including nature of reaction |
| **Family history** | Describe any family history of spontaneous preterm birth (patient herself or siblings) |
| **Social history** | Substance exposure risk including tobacco or cannabis smoking.  Living circumstances, support network  Employment & physical activity required |
| **Examination** | |
| **At bedside** | Body Mass Index calculation using weight and height |
|  | Basic vital signs including blood pressure |
|  | Urinalysis and send MSU |
|  | Examination of periodontal health |
| **Trans-cervical ultrasound** | If formal gynaecology ultrasound report not available  Examine uterine position (anteverted / retroverted) |
|  | Measure cervical length – three conservative measurements and use average length via technique by a provider with adequate training |
| **Vaginal Examination** | Take High Vaginal Swab and Endocervical Swab for exclusion of Bacterial vaginosis and STIs |
|  | If short cervix <25mm noted on ultrasound, examine length of cervix in vagina to ascertain feasibility of cerclage using speculum or digital examination. |

**Limitations of transvaginal cervical length pre-pregnancy**

The lack of evidence and more limited value of pre-pregnancy cervical length should be discussed with the patient. There is no data to support performing pre-pregnancy cervical length in a low risk patient. However, if a cervix is short pre-pregnancy, it is generally accepted to be short during pregnancy. Thus, when taken in context of a previous preterm birth or extensive previous cervical surgery, an anticipated prevention strategy can be discussed with the patient after discussion of the evidence limitations. Criteria, management, and outcome data from preconceptual counselling consultations should be recorded and audited regularly. Transvaginal cervical sonography should be performed by provider with sufficient training and accreditation.

**Formation of Preterm Birth Prevention Plan**

Based on assessment, women will be deemed to be relatively low risk, moderate or high risk of preterm birth in a future pregnancy and can be managed accordingly. Factors important for decision making included significant history of PTB/MTL and cervical length on transvaginal ultrasound. Women with a history of prior MTL, perinatal death resulting from sPTB before 28 weeks or recurrent sPTB are deemed particularly high-risk, with the first two criteria also experiencing the trauma associated with perinatal loss. This is a major factor in subsequent pre-pregnancy decision-making. At pre-pregnancy consultation, four management options are outlined to the patient, and joint decision is made based on individualized risk assessment of sPTB. Management options include:

1. Preterm birth surveillance alone
2. Preterm birth care with progesterone (if history of sPTB or cervical length <25mm in pregnancy) and consideration for cerclage if cervical length <25mm on serial cervical scanning
3. Preterm birth care with elective cervical cerclage in early pregnancy, based on history and pre-pregnancy cervical length
4. Abdominal cerclage pre-pregnancy

**Counselling on Pre-pregnancy Abdominal Cerclage**

Prior to pursuing a trans-abdominal cerclage, a frank discussion should take place with the patient discussing the implications of abdominal cerclage. The risks of pregnancy loss, intra-uterine demise or preterm labour are not common in pregnancies with trans-abdominal cerclage in situ but carry significant risk to the health of the woman if they do occur. Risks to discuss in counselling include:

- Operative risk of the abdominal cerclage placement including bleeding, visceral injury and urinary retention and entry-related risk of laparoscopy
- In the event of second trimester fetal in-utero demise or complication, a serious adverse risk profile is anticipated. Risk includes technical challenge of uterine evacuation in mid-pregnancy including uterine injury, and maternal complications including haemorrhage, retained products of conception and maternal sepsis.
- If intra-uterine demise occurs after viability, delivery by caesarean section is necessary, which may carry elevated risk of haemorrhage, venous thromboembolism along with the technical challenges of delivery a demised fetus at a preterm gestation with an incompletely formed lower segment. Exact risk is difficult to qualify given infrequent occurrence of intra-uterine demise in abdominal cerclage cohort studies.
- All these scenarios have risk of morbidity for the uterus due to technical challenges of hysterotomy at peri-viable gestations, and risk of long-term pelvic morbidity and infertility in setting of maternal and pelvic sepsis.

Decision to proceed with abdominal cerclage should reflect the indication and its’ associated evidence base, the risks associated with adverse pregnancy outcome if it occurs, and the patients’ personal circumstances, ideal family size and plans for future pregnancy.
